# Redox Regulation of Heart Regeneration: An Evolutionary Tradeoff

**DOI:** 10.3389/fcell.2016.00137

**Published:** 2016-12-15

**Authors:** Waleed M. Elhelaly, Nicholas T. Lam, Mohamed Hamza, Shuda Xia, Hesham A. Sadek

**Affiliations:** Department of Internal Medicine, Division of Cardiology, and Hamon Center for Regenerative Science and Medicine, The University of Texas Southwestern Medical CenterDallas, TX, USA

**Keywords:** evolutionary tradeoff, heart regeneration, metabolism, redox regulation, reactive oxygen species (ROS)

## Abstract

Heart failure is a costly and deadly disease, affecting over 23 million patients worldwide, half of which die within 5 years of diagnosis. The pathophysiological basis of heart failure is the inability of the adult heart to regenerate lost or damaged myocardium. Although limited myocyte turnover does occur in the adult heart, it is insufficient for restoration of contractile function (Nadal-Ginard, 2001; Laflamme et al., 2002; Quaini et al., 2002; Hsieh et al., 2007; Bergmann et al., 2009, 2012). In contrast to lower vertebrates (Poss et al., 2002; Poss, 2007; Jopling et al., 2010; Kikuchi et al., 2010; Chablais et al., 2011; González-Rosa et al., 2011; Heallen et al., 2011), adult mammalian heart cardiomyogenesis following injury is very limited (Nadal-Ginard, 2001; Laflamme et al., 2002; Quaini et al., 2002; Bergmann et al., 2009, 2012) and is insufficient to restore normal cardiac function. Studies in the late 90s elegantly mapped the DNA synthesis and cell cycle dynamics of the mammalian heart during development and following birth (Soonpaa et al., 1996; Soonpaa and Field, 1997, 1998), where they showed that DNA synthesis drops significantly around birth with low-level DNA synthesis few days after birth. Around P5 to P7, cardiomyocytes undergo a final round of DNA synthesis without cytokinesis, and the majority become binucleated and exit the cell cycle permanently. Therefore, due to the similarities between the immature mammalian heart and lower vertebrates (Poss, 2007; Walsh et al., 2010), it became important to determine whether they have similar regenerative abilities. Recently, we demonstrated that removal of up to 15% of the apex of the left ventricle of postnatal day 1 (P1) mice results in complete regeneration within 3 weeks without any measurable fibrosis and cardiac dysfunction (Porrello et al., 2011). This response is characterized by robust cardiomyocyte proliferation with gradual restoration of normal cardiac morphology. In addition to the histological evidence of proliferating myocytes, genetic fate-mapping studies confirmed that the majority of newly formed cardiomyocytes are derived from proliferation of preexisting cardiomyocytes (Porrello et al., 2011). More recently, we established an ischemic injury model where the left anterior descending coronary artery was ligated in P1 neonates (Porrello et al., 2013). The injury response was similar to the resection model, with robust cardiomyocyte proliferation throughout the myocardium, as well as restoration of normal morphology by 21 days. However, this regenerative capacity is lost by P7, after which injury results in the typical cardiomyocyte hypertrophy and scar-formation characteristic of the adult mammalian heart. Not surprisingly, the loss of this regenerative capacity coincides with binucleation and cell cycle exit of cardiomyocytes (Soonpaa et al., 1996; Walsh et al., 2010). An important approach toward a deeper understanding the loss of cardiac regenerative capacity in mammals is to first consider ***why***, and not only ***how***, this happens. Regeneration of the early postnatal heart following resection or ischemic infarction involves replacement of lost myocardium and vasculature with restoration of normal myocardial thickness and architecture, with long-term normalization of systolic function. Why would the heart permanently forego such a remarkable regenerative program shortly after birth? The answer may lie in within the fundamental principal of evolutionary tradeoff.

## Evolutionary tradeoffs

**Figure 1:**
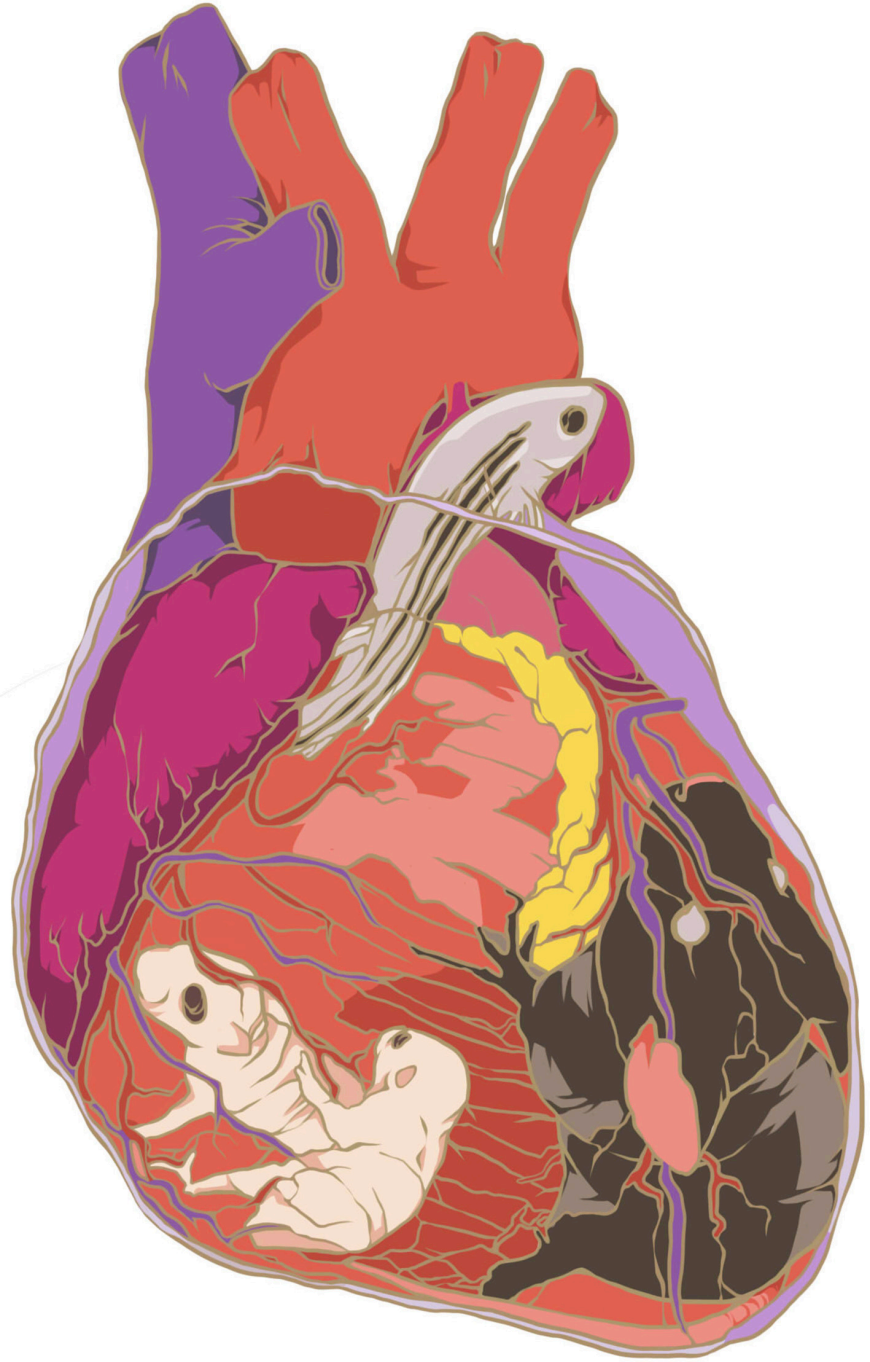
**Artistic view of heart regeneration models (kindly provided by Usuma Thet)**.

Darwin (1859) in *The Origin of Species* stated that “The whole organism is so tied together that when slight variations in one part occur, and are accumulated through natural selection, other parts become modified. This is a very important subject, most imperfectly understood.” He captured the concept of evolutionary tradeoff perfectly by saying that “in order to spend on one side, nature is forced to economize on the other side.” This concept may hold the key to understanding why the adult mammalian heart does not regenerate, while the fetal and neonatal hearts can (Figure 1). The sequence of events that occur after birth, where the mammalian heart becomes the most energy demanding organ, are compelling enough to support the hypothesis that the loss of cardiac regeneration in adult mammals is a form of evolutionary tradeoff.

## Examples of evolutionary tradeoffs

In general, there are two types of evolutionary tradeoff; namely one or two-trait tradeoffs. One trait evolutionary tradeoffs, which are the focus of the current article, can be caused by opposing selections resulting from different environments, manifesting as an apparent cost of a certain functional trait (Agrawal et al., 2010).

Fossil records of *Waimanu* (ancestor of penguins) estimates the divergence of penguins from other birds began approximately 61–62 million years ago (Slack et al., 2006). The wing length (relative to body size) of *Waimanu* was shorter than general birds, but longer than penguins (Slack et al., 2006). Following the structural evolutional transition of wings to adapt for swimming, wing feathering in an early penguin (*Inkayacu*) fossil from approximately 56.3 million years ago suggests evolutionary changes to became far more hydrodynamic (Clarke et al., 2010). In modern day penguins, evolutionary tradeoffs which occurred for wings to become biomechanically more efficient for swimming, resulted in wings which became far less efficient for flying (Elliott et al., 2013).

Another example is manifested in horse limbs evolution. One important tradeoff resulted from the transition of horses from the previous digitigrade (walking on digits) to unguligrade (hoofed) animals. The digits of ancestors of the horse allowed it to maneuver on complex surfaces, which translated into greater agility, while the later hoof was superior for running long distances on flat surfaces, which translated into greater speed and endurance. With the development of hoofs, horses lost much of their agility and their ability to browse and maneuver in confined spaces. As a tradeoff, however, they gained great speed and endurance, which was probably needed to escape predators (Forsten, 1989).

## Is loss of heart regeneration in mammals an evolutionary tradeoff?

To determine whether the loss of the endogenous cardiac regeneration ability of the mammalian heart is a form of evolutionary tradeoff, we must carefully consider certain aspects of mammalian heart evolution, the events that occur at the time of cell cycle arrest of postnatal mammals, and the mechanism of cardiomyocyte turnover in the adult heart.

### Evolution of the mammalian heart

The vertebrate heart has undergone a significant degree of evolutionary changes. In the earliest vertebrates, the heart was a low-pressure contractile vessel, which later developed into a two-chamber heart in early fish. Further evolution in fish, amphibians, birds and mammals later resulted in septation, chamber separation and marked thickening of the ventricles with increased ventricular pressure. In addition to the morphological changes, cardiac evolution was associated with a several fold increase in heart rate, which is needed to sustain high cardiac output. Therefore, the mammalian heart evolved from a single low-pressure tube, to the complex, high-pressure, high heart rate, multi-chamber organ of adult mammals.

In the evolution literature, the concept of space constraint is hypothesized as a major determinant of loss of the ability of cardiomyocytes to divide. While this may certainly be a factor, it does not explain the permanent loss of cardiomyocyte proliferative ability. In other words, space constraint may in fact stop cardiomyocyte proliferation, but why would it prevent any ability of cardiomyocytes to divide even after substantial cardiomyocyte loss? Another argument against that concept is the fact that the mammalian heart increases in size during postnatal development by several folds through hypertrophic cardiomyocyte growth. This occurs following the permanent cell cycle arrest of cardiomyocytes, and therefore argues against the space constraint hypothesis. In other words, if the heart still has to grow several folds during postnatal development, why does it do so through hypertrophic rather than hyperplastic growth?

### Oxygenation, metabolism, and myocardial regeneration

One of many factors shared by organisms that are capable of heart regeneration is the oxygenation state. For example, the zebrafish's stagnant and warm aquatic environment has 1/30th oxygen capacitance compared to air, and is prone to poor oxygenation (Rees et al., 2001; Roesner et al., 2006). Moreover, the zebrafish circulatory system is relatively hypoxemic, as it has a primitive two-chambers heart, which results in mixing of arterial and venous blood. Similarly, the mammalian fetal circulation is shunt-dependent with significant mixing of arterial and venous blood. Although blood in the umbilical vein going to the fetus is 80–90% saturated with a PaO_2_ of 32–35 mmHg, the blood ejected from the left ventricle is only 65% saturated with a PaO_2_ of 25–28 mmHg (Dawes et al., 1954), which is quite hypoxemic compared to the postnatal circulation with a saturation above 95% and a PaO_2_ of 100 mmHg. Therefore, both the zebrafish and mammalian fetal heart reside in relatively hypoxic environments; however transition from embryonic- to postnatal-circulation soon after birth drastically changes the oxygenation state of cardiomyocytes (Puente et al., 2014). In parallel to the oxygenation state, energy metabolism of the embryonic and adult heart is quite distinct. During embryonic development, when cardiomyocytes rapidly proliferate, the relatively hypoxic embryonic heart utilizes anaerobic glycolysis as a main source of energy (Fisher et al., 1980; Lopaschuk et al., 1992), whereas adult cardiomyocytes utilize the oxygen-dependent mitochondrial oxidative phosphorylation as an energy source (Wisneski et al., 1985; Gertz et al., 1988).

### Oxidative stress, DNA damage response (DDR) pathway in postnatal mouse heart and cell cycle regulation

The energy advantage of mitochondrial oxidative phosphorylation over glycolysis is not without deleterious consequences, as the mitochondrion is considered the major source of free radical production (Harman, 1972; Nohl and Hegner, 1978; Miquel et al., 1980; Turrens, 1997, 2003). Our studies show that there is a significant increase in ROS in cardiomyocytes from day 1 to day 7 after birth. Mitochondrial ROS are generated as a consequence of electron leak by the electron transport chain (Rudolph and Heyman, 1974; Koopman et al., 2010) and can cause cellular toxicity by promoting damage of proteins, lipids or DNA, such as oxidized base, single- or double-strand breaks, resulting in cell cycle arrest, apoptosis or cellular senescence (Moos et al., 2000; Marnett et al., 2003; Hoeijmakers, 2009). We hypothesize that oxidative DNA damage might increase in cardiomyocytes postnatally and play a role in postnatal cell cycle arrest. In order to test this, we assessed oxidative base modification of DNA and showed that one of the oxidized bases in DNA, 8-oxo-7,8-dihydroguanine (8-oxoG), was undetectable at p0, but significantly increased at P7 and P14. In concert, the DNA damage response (DDR) pathway was also significantly activated as indicated by upregulation of Ser1981 phosphorylated-Ataxia Telangiectasia Mutated (p-ATM) kinase, an upstream kinase that activates multiple components of the DDR pathway, at P7 and P14. These results demonstrate that the increase in mitochondrial respiration corresponds temporally with an increase ROS in the neonatal heart and activation of DDR. Our published results also suggest that mitochondrial ROS mediated activation of the DDR is an important upstream event that mediates cell cycle arrest of postnatal cardiomyocytes, where mitochondrial catalase (mCAT) overexpression in cardiomyocytes decreases oxidative DNA damage and prolongs the postnatal window of cardiomyocyte proliferation (Puente et al., 2014). However, the factors that mediate increased mitochondrial ROS production and the subsequent oxidative DNA damage are not well understood.

### Cardiomyocyte turnover in the adult mammalian heart

Recent studies using data from the nuclear fallout during the cold war by using ^14^C dating to measure cardiomyocyte age indicated that turnover rates of human cardiomyocytes are about 1% per year at the age of 25 (Bergmann et al., 2009). These rate further decrease to 0.45% around 75 years of age, and approximately 45% of cardiomyocytes are replaced over the normal human lifespan (Bergmann et al., 2009). Importantly, a recent study described the dynamics of cardiomyocyte turnover in the adult mammalian heart using a novel multi isotope mass spectrometry (MIMS), and showed that the newly formed cardiomyocytes are derived from preexisting cardiomyocytes rather than a progenitor or stem cell population (Senyo et al., 2013). Intriguingly, the rate of cardiomyocyte turnover is similar to what has been previously described in humans (Bergmann et al., 2009).

Since cell cycle exit of cardiomyocytes shortly after birth is mediated by an increase in mitochondrial-derived ROS leading to oxidative DNA damage, we hypothesized that the population of cycling cardiomyocyte in the adult heart is protected from oxidative DNA damage by residing in hypoxic microenvironments, not unlike other cycling cells in the hematopoietic stem cell niche, or the hippocampus of the brain. In these hypoxic niches, stabilization of hypoxia-inducible factor-1 alpha (Hif-1α) is critical for their maintenance and function. Therefore, we reasoned that stabilization of Hif-1α identifies cycling cardiomyocytes within the uninjured adult heart. We generated a transgenic mouse that expresses a fusion protein where the oxygen dependent degradation (ODD) domain of Hif-1α is fused to the tamoxifen inducible CreERT2 under the cardiomyocyte-specific MHC promoter, thereby fate mapping hypoxic cardiomyocytes and their progeny after tamoxifen administration. Shortly after the tamoxifen pulse, we identified a rare population of hypoxic cardiomyocytes that contributed widely to new cardiomyocyte formation in the adult heart. Intriguingly, these hypoxic cardiomyocytes displayed characteristics of fetal/neonatal cardiomyocytes such as smaller cell size, mononucleation and absence of oxidative DNA damage. These findings support the hypothesis that cardiomyocyte turnover in the postnatal mammalian heart is inhibited by oxidative metabolism and the resulting oxidative DNA damage.

## Conclusion

Evolutionary tradeoffs can manifest as a specific cost (loss or gain) of a functional trait. Current evidence suggests that loss of the regenerative ability of the postnatal mammalian heart might be a manifestation of a one-trait evolutionary tradeoff, where the cost of higher energy efficiency is loss of cardiomyocyte proliferation. Conceptually, this has important implications for cardiac regeneration therapies aimed at inducing adult cardiomyocyte proliferation. For example, it may not be possible to regenerate the adult mammalian heart without either profoundly altering the redox state of cardiomyocytes, or foregoing at least some of the energy advantage provided by mitochondrial respiration.

## Funding

This work is supported by a grant from the Hamon Center for Regenerative Science and Medicine, University of Texas Southwestern Medical Center. HS is supported by National Institute of Health grant 1R01HL115275-01. NL is supported by a Sir Keith Murdoch Australia to US Fellowship from the American Australian Association.

## Author contributions

WE, SX, MH, and NL researched the topic and helped write the manuscript. HS conceived the hypothesis and edited the manuscript.

### Conflict of interest statement

The authors declare that the research was conducted in the absence of any commercial or financial relationships that could be construed as a potential conflict of interest.
